# Assessing the Potential of Generative Artificial Intelligence Models to Assist Experts in the Development of Pharmacokinetic Models

**DOI:** 10.34172/apb.025.43852

**Published:** 2025-06-03

**Authors:** Sergio Sánchez-Herrero, Laura Calvet Liñan

**Affiliations:** ^1^Department of Computer Science, Multimedia and Telecommunication, Universitat Oberta de Catalunya, Barcelona, 08018, Spain; ^2^Department of Telecommunications & Systems Engineering, Universitat Autònoma de Barcelona, Sabadell, 08202, Spain

**Keywords:** Artificial intelligence, Generative models, Pharmacokinetics, ChatGPT, Modelling

## Abstract

**Purpose:**

This study explores the potential of generative AI models to aid experts in developing scripts for pharmacokinetic (PK) models, with a focus on constructing a two-compartment population PK model using data from Hosseini et al.

**Methods:**

Generative AI tools ChatGPT v3.5, Gemini v2.0 Flash and Microsoft Copilot free could help PK professionals— even those without programming experience—learn the programming languages and skills needed for PK modeling. To evaluate these free AI tools, PK models were created in R Studio, covering key tasks in pharmacometrics and clinical pharmacology, including model descriptions, input requirements, results, and code generation, with a focus on reproducibility.

**Results:**

ChatGPT demonstrated superior performance compared to Copilot and Gemini, highlighting strong foundational knowledge, advanced concepts, and practical skills, including PK code structure and syntax. Validation indicated high accuracy in estimated and simulated plots, with minimal differences in clearance (Cl) and volume of distribution (V c and V p) compared to reference values. The metrics showed absolute fractional error (AFE), absolute average fractional error (AAFE), and mean percentage error (MPE) values of 0.99, 1.14, and -1.85, respectively.

**Conclusion:**

These results show that generative AI can effectively extract PK data from literature, build population PK models in R, and create interactive Shiny apps for visualization, with expert support.

## Introduction

 Generative artificial intelligence (AI) models, including ChatGPT from OpenAI, Bard from Google (Gemini), and Copilot from Microsoft, exemplify a rapidly advancing technology that has garnered significant private and public attention.^1^ These models possess the potential to transform pharmacokinetics (PK) by offering novel research and analysis pathways. However, it is imperative to acknowledge that these technologies are still in the early stages of development and are susceptible to misuse. It is imperative to ensure meticulous integration into PK research.

 PK and pharmacodynamics (PD) modelling play a crucial role in preclinical research by enabling the characterization of drug concentration-time profiles, interspecies scaling, and dose selection. Population-based approaches and mathematical models are essential tools that help pharmacologists assess drug exposure, efficacy, and safety. The use of nonlinear mixed-effects and Bayesian methods has improved the analysis of limited clinical data, helping to predict variability, optimise dosing, and support regulatory decisions.^2^

 Recent advancements, particularly those stemming from machine learning (ML) and generative models, have led to novel approaches for enhancing PK modelling. These approaches involve the generation of synthetic data that emulates real-world conditions and the simulation of intricate biological systems.^3^ When integrated with domain expertise, these models have the potential to enhance prediction accuracy, uncover latent patterns, and facilitate decision-making processes in the field of drug development.^4^ The utilisation of tools such as pyDarwin, which integrates ML with NONMEM, exemplifies the efficacy of hybrid methodologies.^5^ However, its limitations – particularly in relation to complex or unfamiliar tasks – mean that it must be used with caution, validated rigorously, and applied by experts in PK and statistics alongside traditional methods to ensure reliability.^6^

 In this context, the objective of this study is to provide a preliminary exploration of the development of generative AI, with a particular focus on examining the capabilities of ChatGPT, Copilot, and Gemini in assisting PK experts by providing simulation support in code and generating relevant knowledge for practice, learning, and research. The focus of this exploration will be on examining these AI generative models in providing simulation assistance in code and generating knowledge relevant to the practice, learning and research of experts in PK. The following two points will be analysed in this study: i) the potential of generative AI models to act as tools to assist PK experts in the execution of their professional duties; and ii) the presentation of an illustrative example by validating a population analysis developed by Hosseini et al.^7^

## Material and Methods

###  Software

 Different generative AI models were used like ChatGPT v3.5 (https://chat.openai.com; OpenAI; version as of April 4, 2024),Gemini v2.0 Flash (https://gemini.google.com; Google AI; version as of December 11, 2024),and Microsoft Copilot free (https://copilot.microsoft.com; Microsoft; version as of September 21, 2023).It is important to take into account that all generative AI models are free.

 R program language (version 4.2.1) was used in the current study for data pre-processing and pop analysis implementation. The following packages were utilized: ggplot2 (version 3.5.0),^8^ readxl (version 1.4.1),^9^ Shiny (version 1.7.2)^10^ and deSolve (version 1.40).^11^ Unless otherwise specified, default parameters were used for each programming function.

**Figure 1:**
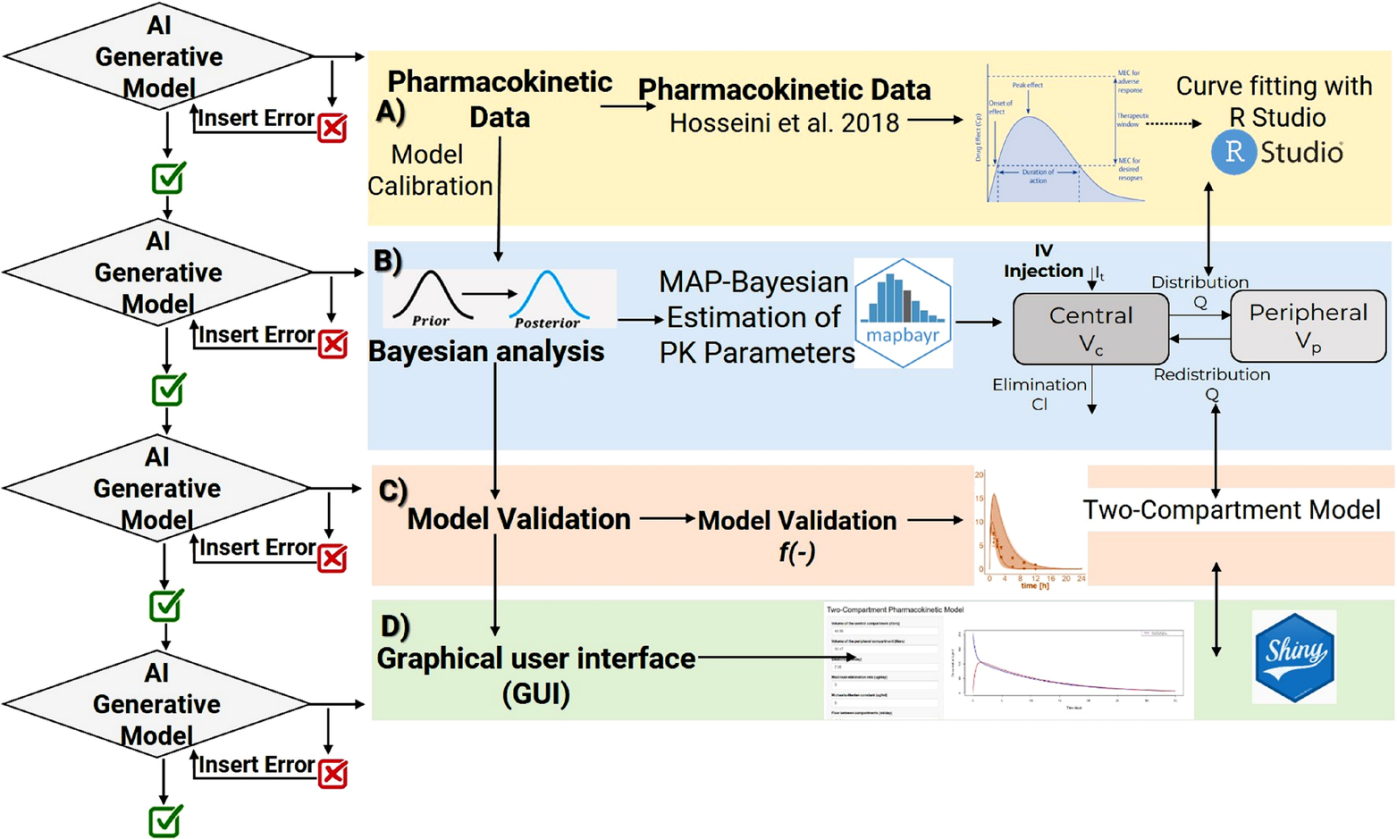
Workflow followed to build a generative AI application for developing a two-compartment model. AI generative model developed and designed to process and generate text in response to user queries and requests. A) Pharmacokinetic data by Hosseini et al.^7^ B) Two-Compartment model was calibrated with experimental datasets and was optimized and parameter uncertainty and variability were characterized within a MAP-Bayesian estimation of PK parameters framework (mapbayr).^12^ C) The final model was subjected to evaluation/validation. D) The final PK population model was converted to a web-based graphical user interface with Shiny R package^10^

 AI generative models were employed to provide descriptions and responses to queries according to the workflow outlined in Figure 1. Utilizing the generated R code, subsequent inquiries were made to enhance the functionalities of the PK model.

###  Pharmacokinetics model for simulation

 To evaluate the use of generative AI in pharmacokinetic modelling, we replicated the two-compartment model from Hosseini et al^7^ (Case 1) using R; dataset and details are available in the original Supplementary material.

###  Data fitting re-estimation

 PK parameters were estimated using the mapbayr package in R (v0.10.0), which applies a maximum a posteriori Bayesian method to models built with mrgsolve (version 1.4.1).^12^ Initial values are listed in Table 1.

**Table 1:** Initial conditions for the two-compartment model

**Variable**	**Estimated**	**Initial**	**Units**	**Min**	**Max**
V_1_	True	40	mm/kg	10	200
V_2_	True	40	mm/kg	10	200
C_l_	True	5	mm/day/kg	1	30
Q	True	10	mm/day/kg	1	100
V_m_	False	0	µg/day/kg	0	120
K_m_	False	5	µg/mm	0.1	100

###  Model Validation

 The resulting code was executed to predict two-compartment model parameters, which were then compared to reference values from Hosseini et al.^7^ For reproducibility and verification, prediction performance was assessed using standard metrics: prediction error (PE), mean percentage error (MPE), average-fold error (AFE), and absolute average-fold error (AAFE). Predictive performance was considered satisfactory if AFE and AAFE values fell within the 0.8–1.25-fold range.^13^

 A visual predictive check (VPC) was conducted to assess the predictive performance of the PK model. Observed concentrations were dose-normalized to a standard dose using a Monte Carlo simulation approach implemented in R, incorporating generative models along with the parameter estimates and variability described in Table 2.

**Table 2:** Common values and variability of model parameters for the simulated data

	**Simulated data two-compartment model** **(PIs 5th and 95**^th^**)**
Dose	10 mg/100 mg
Population	1000 subjects
Population parameters	*V c*= *fit l* *V p*= *fit l* *CLt*= *fit l/h* *CLd*= *fit l/h*
Inter-subject variability	variability population result
Residual error	*V ar*= (0.01 + 0.1 · *IPRED*)^2^

 Additionally, goodness-of-fit (GOF) plots were performance to assess model adequacy. R can generate a variety of GOF plots, including graphs like observations versus individual and population model predictions or ETA distribution from estimation results.

###  GitHub repository

 All steps, data analysis procedures, results, and accompanying analyses presented in this study are made openly accessible through an open repository for the sake of transparency, reproducibility, and community engagement. The URL for the guide is: https://github.com/sersanchezherrero/Generative_AI_PKpop_Model/tree/main.

## Results

###  Two-compartment model using generative model

 Various generative AI tools (Copilot, Gemini and ChatGPT) were prompted to generate a two-compartment model based on Hosseini et al.^7^ Outputs were compared to published simulations and real data. All prompts, steps and code are available at https://github.com/sersanchezherrero/Generative_AI_PKpop_Model/tree/main.

**Figure 2:**
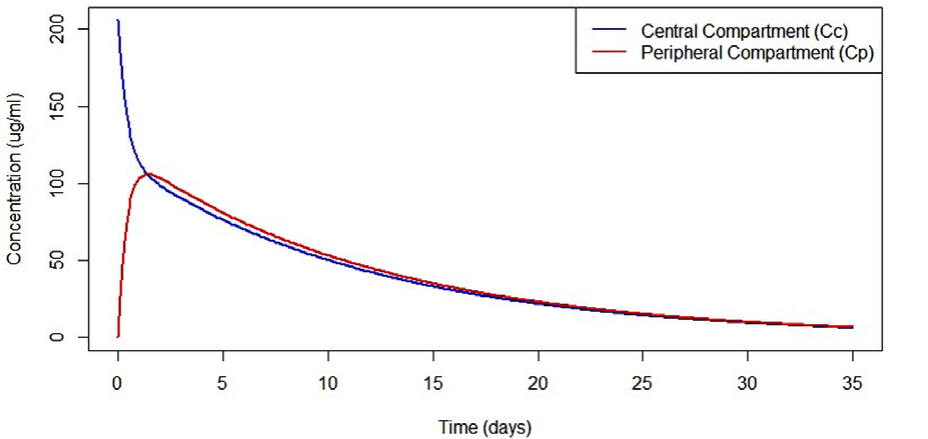
Two-Compartment pharmacokinetics generated from the generative AI code located in https://github.com/sersanchezherrero/Generative_AI_PKpop_Model/blob/main/R/two_compartment_model.R. Blue line central compartment concentration. Red line peripheral compartment concentration. The two-compartment model follows the expected pharmacokinetic behaviour, demonstrating proper distribution and elimination phases

 Each generative AI model produced a slightly different R code for developing the PK model, leading to variations in the results. We encountered several errors while executing the generated R codes, which were resolved by sharing the issues with the respective AI models. Gemini and Microsoft Copilot did not produce successful results and they were excluded from further steps. It is important to note that specificity in input generally yields better results. However, discrepancies across AI models were mainly related to the graph legends. The text input used to obtain Figure 2 from the PK model is available at https://github.com/sersanchezherrero/Generative_AI_PKpop_Model/blob/main/generative_AI_text/model.txt

 The same code and asking text were adapted by AI generative models using the Shiny package to create a user interface. The additional text provided to the AI models was: “*The code you generate, I want it in Shiny, with inputs for the parameter definition and the time”*. R code to obtain Shiny application is located in https://github.com/sersanchezherrero/Generative_AI_PKpop_Model/blob/main/R/shinyall.R.

**Figure 3:**
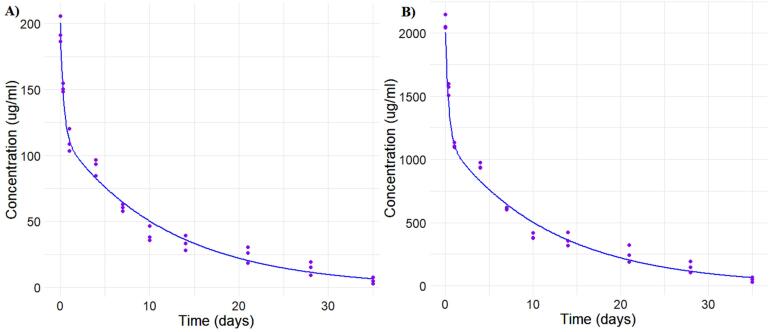
Pooled fit graphs generated with from generative AI model code generated by dose according to intravenous administration, (A) 10 mg. and (B) 100 mg. Blue: Simulated fit data. Purple: Real data. Concentration-time profiles (A-B) demonstrate consistent pharmacokinetic trends across observed times, with smooth curves indicating stable model behaviour and reliable predictions

**Figure 4:**
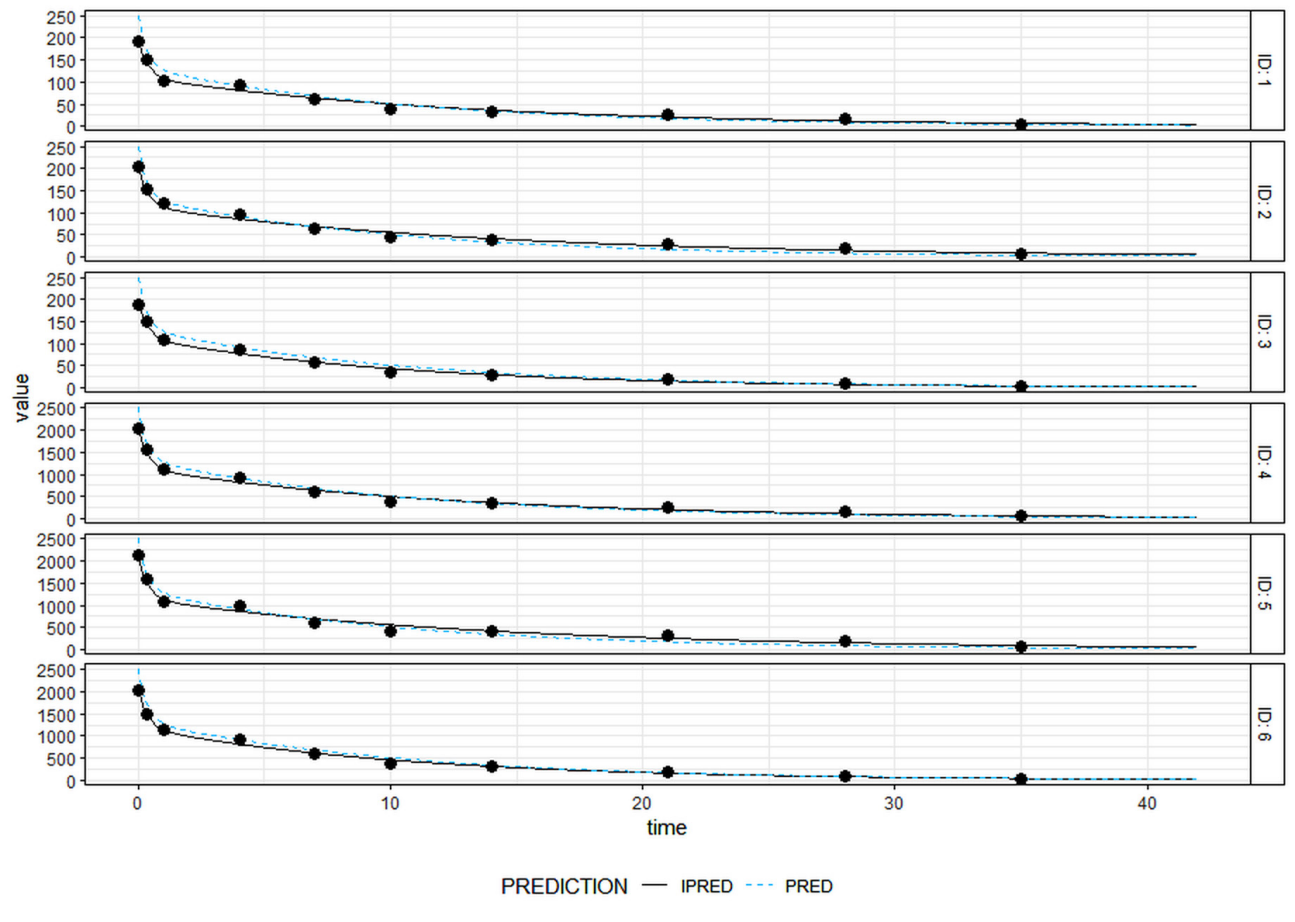
Individual concentrations and individual predicted curves for the pharmacokinetics models generated by mapbayr package. Black dots are real data. Black lines are individual predictions (IPRED). Dash-blue lines are population predictions (PRED). On the right of the figure are located ID patient. The consistent overlap of trajectories across subjects indicates reproducible pharmacokinetic behaviour and model reliability

 Parameter estimation for population analysis was most straightforward using mapbayr, as generative AI models readily interpreted its Bayesian framework.^12^ The results of the pooled fitting are illustrated in Figure 3 for 10 mg and 100 mg, and the parameter estimates for both pooled, Figure 4 for individual mapbayr predicted curves and the group specific fittings are listed in Table 3, demonstrating relatively low standard errors. The steps followed to obtain the results are available at https://github.com/sersanchezherrero/Generative_AI_PKpop_Model/blob/main/generative_AI_text/pop.txt.

**Table 3:** Fitting combined pooled results Hosseini et al^7^ versus PhysPK

**Name**	**Initial**	**Fit-group**	**AI generative Model**	**Units**
V1	40	49.15 + /-3.8	49.86 + /-1.43	ml/kg
V2	40	34.61 + /-5.2	33.14 + /-1.33	ml/kg
CL	*5*	*6.89 + /-0.2*	*7.02 + /-0.78*	ml/day/kg
Cld	10	45.5 + /-17.4	50.55 + /-0.5	ml/day/kg

**Figure 5:**
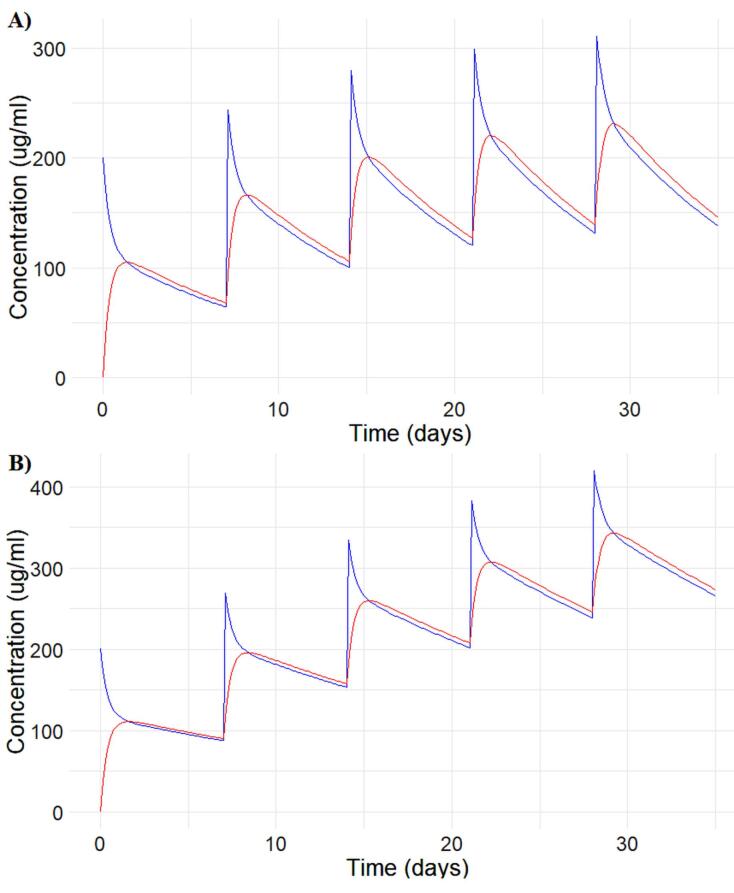
Two-Compartment pharmacokinetic model projection for the PK profile of a 10 mg/kg 4qw (weekly dosing for 4 weeks) IV dosing regimen under A) CL1 = 7.02 (Fit) and B) CL2 = 0.5 x Cl1 = 3.51. Blue line: Central Compartment. Red line: Peripheral Compartment. The smooth concentration-time trajectories (A-C) reflect reliable model performance, with no aberrant patterns, indicating stable parameter estimation and predictive accuracy

 On the other hand, the model and parameter estimates were used together to project PK for an alternate dosing regimen and explore potential PK variability. The parameter estimates from the two-compartment model were saved to use them for projecting the PK profile of a 10 mg/kg 4qw (weekly dosing for 4 weeks) IV dosing regimen under different conditions, listed in Table 4. The population simulation based on variably exposed in Table 4 shows AUC (0-28) similar to the reference too. Figure 5 provides a summary of the projective simulations. Prompts text could be located in https://github.com/sersanchezherrero/Generative_AI_PKpop_Model/blob/main/generative_AI_text/simulation.txt and R code in https://github.com/sersanchezherrero/Generative_AI_PKpop_Model/blob/main/R/simu.R.

**Table 4:** Fitting combined pooled results Hosseini et al^7^ versus PhysPK

	**Scenario**	**Ref. AUC (0-28)**	**AI AUC (0-28)**
Simulation	CL1 = 7.02 (Fit)	4162.31	4068.77
Simulation	CL2 = 0.5 x Cl1 = 3.51	5759.83	5652.29

###  Visual predictive check

**Figure 6:**
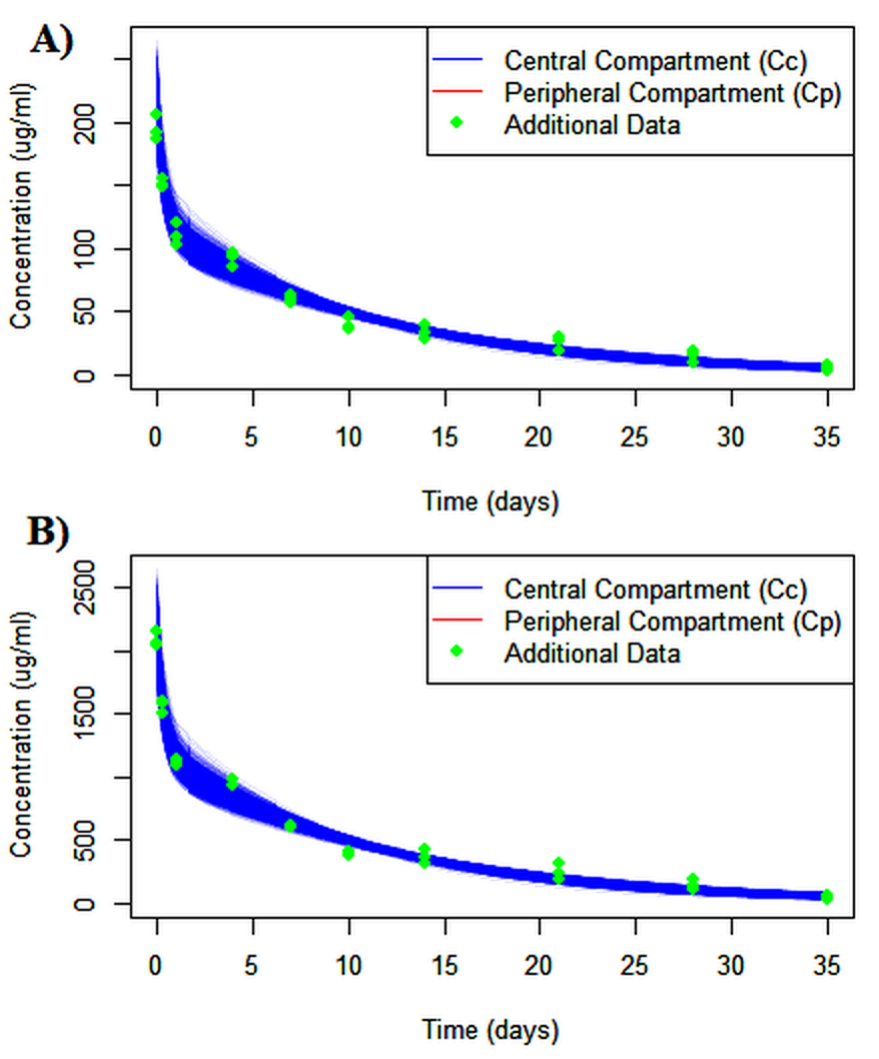
Virtual predicted check (VPC) generated with R by dose according to intravenous administration. A population dataset of 1000 subjects was generated using Monte Carlo simulation method for A) 10 and B) 100 mg administration. The fitted concentration-time curves demonstrate good agreement between the model and observed data

 To evaluate the effectiveness of a model’s predicted performance, VPC charts were interacted with the results, with further details provided at https://github.com/sersanchezherrero/Generative_AI_PKpop_Model/blob/main/generative_AI_text/vpc.txt. The graph generated by VPC in Figure 6 provides a summary of the predictive simulations.

###  Parameter distribution profiles

**Figure 7:**
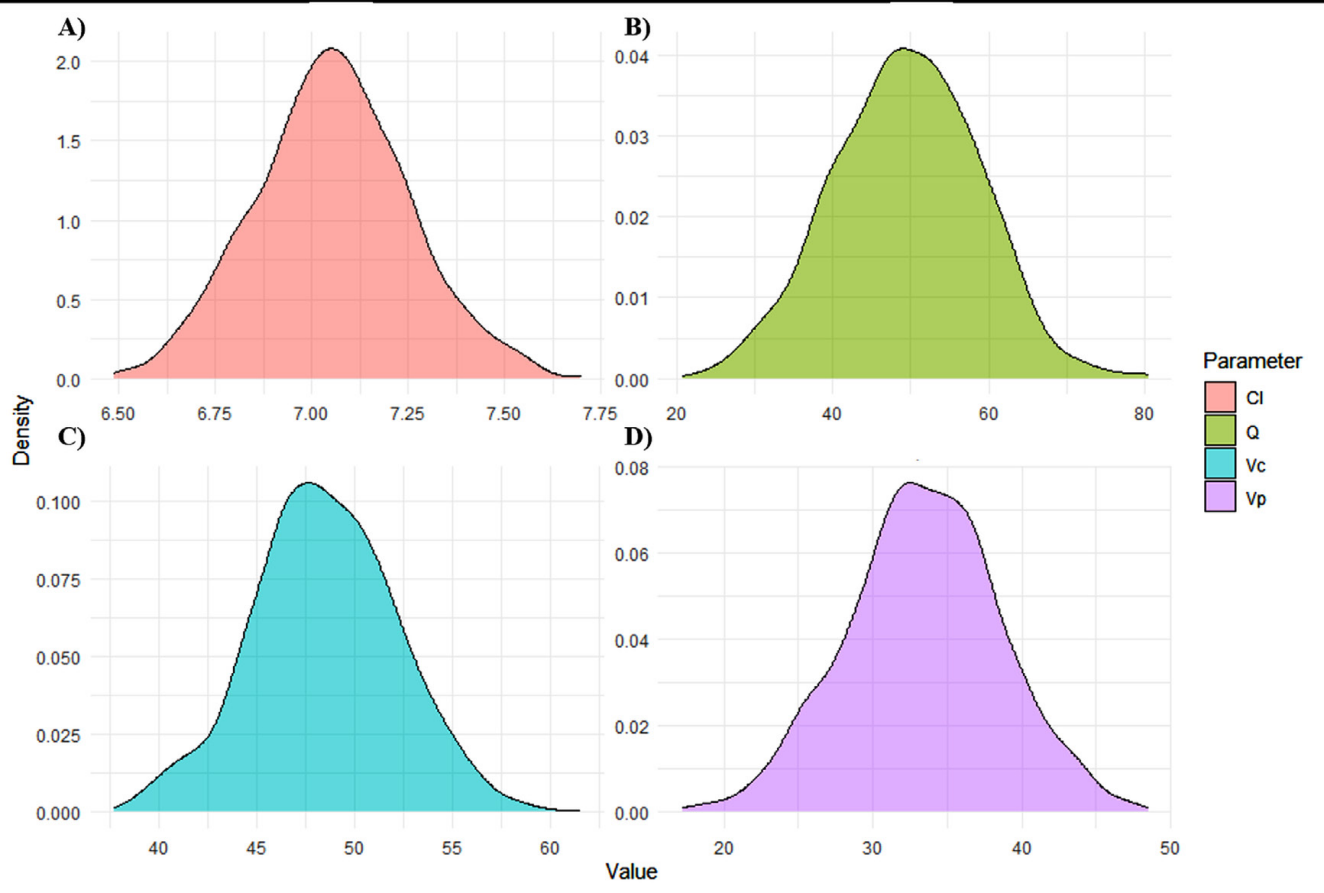
Density graph from Montecarlo simulation parameters. A) Clearance (CL). B) Distribution (Q). Central Volume (Vc). D) Peripheral volume (Vp). The Figure provides fixed estimates for key parameters, supporting quantitative interpretation. Such plots are essential for evaluating parameter precision in Bayesian or population modeling. The smooth, symmetric density curves (A-D) indicate well-behaved parameter distributions, suggesting reliable model estimates

 The log-normal distribution of parameters holds significance as it cannot reveal the presence of subpopulations. In Figure 7, density graphs are reported. They effectively convey information regarding the variability around each parameter, affirming the suitability of the chosen distribution for this model. The generative AI models were interacted with the results, with further details provided at https://github.com/sersanchezherrero/Generative_AI_PKpop_Model/blob/main/generative_AI_text/dis.txt

 To evaluate the accuracy of model-based the AFE and AAFE values fall between 0.8 and 1.25. The precision metrics results were *AFE*= 0.99, *AAFE*= 1.14 and *MPE*= −1.85 for the six real patients data. The metrics showed proper accuracy based on references.^13^

###  Goodness-of-fit

**Figure 8:**
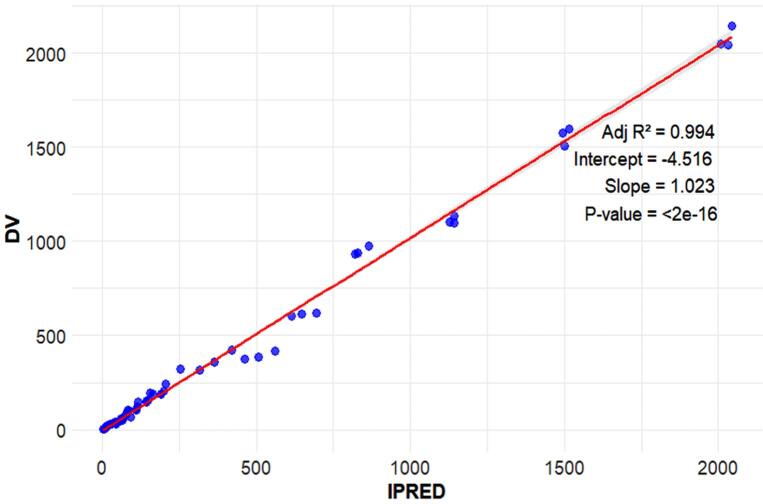
Goodness-of-fit plots (PRED versus DV) for the final model. Abbreviations: DV, observed plasma concentration; iPreD, individual predicted values. Observed vs. Individual Predicted Values (IPRED) with Regression Line. The tight alignment of points along the diagonal line indicates excellent model performance, with predictions closely matching actual observations. This validation step ensures the reliability of the model for interpreting or forecasting outcomes

 AI generative model code for developing GOF plot and ETA distribution plots are located in https://github.com/sersanchezherrero/Generative_AI_PKpop_Model/blob/main/generative_AI_text/gof.txt. Generated plots are shown in Figure 8. Visual inspection of the precision plot (observation vs. individual and population model prediction) indicates that the predictions obtained are acceptable compared to the reference.

## Discussion

 Generative AI models such as ChatGPT and Copilot are emerging as valuable tools in PK modeling. Although not specifically designed for pharmacometrics, they can assist with code generation, model interpretation, and troubleshooting, supporting both experienced users and those with limited programming skills.^14^

 Traditional PK analyses typically rely on software like NONMEM, Monolix, and WinNonlin, which offer advanced functionalities but often require specialized expertise. In contrast, generative AI tools facilitate more accessible and flexible workflows within platforms like R, helping to translate complex PK concepts into executable code and promoting interdisciplinary collaboration.^15,16^

 In our study, we developed a two-compartment population PK model for single-dose intravenous antibody administration using ChatGPT and Copilot. The AI-generated models produced pharmacokinetic parameter estimates with high accuracy. For instance, clearance values (CL and CLd) were 6.89 ± 0.2 and 45.5 ± 17.4, closely matching the reference values of 7.02 ± 0.78 and 50.55 ± 0.5, respectively. Similarly, volumes of distribution (V1 and V2) were estimated at 49.15 ± 3.8 and 34.61 ± 5.2, compared to reference values of 49.86 ± 1.43 and 33.14 ± 1.33. Model performance metrics were also within acceptable ranges, with an AFE of 0.99, AAFE of 1.14, and MPE of –1.85. These results align with previous studies, such as those by Hosseini et al., validating the ability of generative AI to accurately replicate standard population PK models.

 However, these tools have important limitations. Their responses are based on pre-trained data, which may not reflect the latest advancements in pharmacometrics, and they lack the specialized insight of human experts. There is also a risk of generating oversimplified or incorrect outputs, particularly in complex or novel scenarios. Issues related to reproducibility, transparency, and intellectual property must be considered, and ethical use requires clear attribution and expert oversight.

 Despite these challenges, ongoing advances—such as the improved reasoning and language capabilities in models like ChatGPT-4—suggest that generative AI will play an increasingly important role in PK/PD workflows. Used judiciously, these tools can streamline the early stages of model development, improve efficiency, and expand accessibility while maintaining scientific rigor through appropriate validation and human supervision.^17,18^

## Conclusion

 This study highlights the transformative role of AI generative models in pharmacokinetic modelling using R. These tools enhance information synthesis, collaboration, education, and code generation, making PK modeling more accessible and efficient.

 By developing a two-compartment population PK model, we demonstrated that AI tools—particularly ChatGPT and Copilot—can accurately estimate key parameters (CL, V, CLd), with minimal discrepancies compared to traditional approaches. Their integration with R enables streamlined workflows, reduces manual errors, and supports reproducible research.

 AI models offer significant advantages in routine model evaluation, publication preparation, and decision-making. However, they should complement—not replace—human expertise. Responsible use requires clear objectives, context, and expert oversight to ensure scientific rigor.

 In summary, AI generative models represent a major advancement in pharmacometrics, with growing potential to further transform the field through continued innovation and integration.

## Competing Interests

 The authors declare that they have no conflicts interests.

## Data Availability Statement

 All data used, generated or analyzed during this study are included in https://github.com/sersanchezherrero/Generative_AI_PKpop_Model/tree/main.

## Ethical Approval

 Not applicable.

## Supplementary Files


Supplementary File contains a zip file.
