# Supplementary figures and images for: Assessing the Potential of Generative Artificial Intelligence Models to Assist Experts in the Development of Pharmacokinetic Models
Source: Adv Pharm Bull. 2025 Jun 3;15(2):467–73. doi: 10.34172/apb.025.43852 (PMC12413966; doi:10.34172/apb.025.43852)

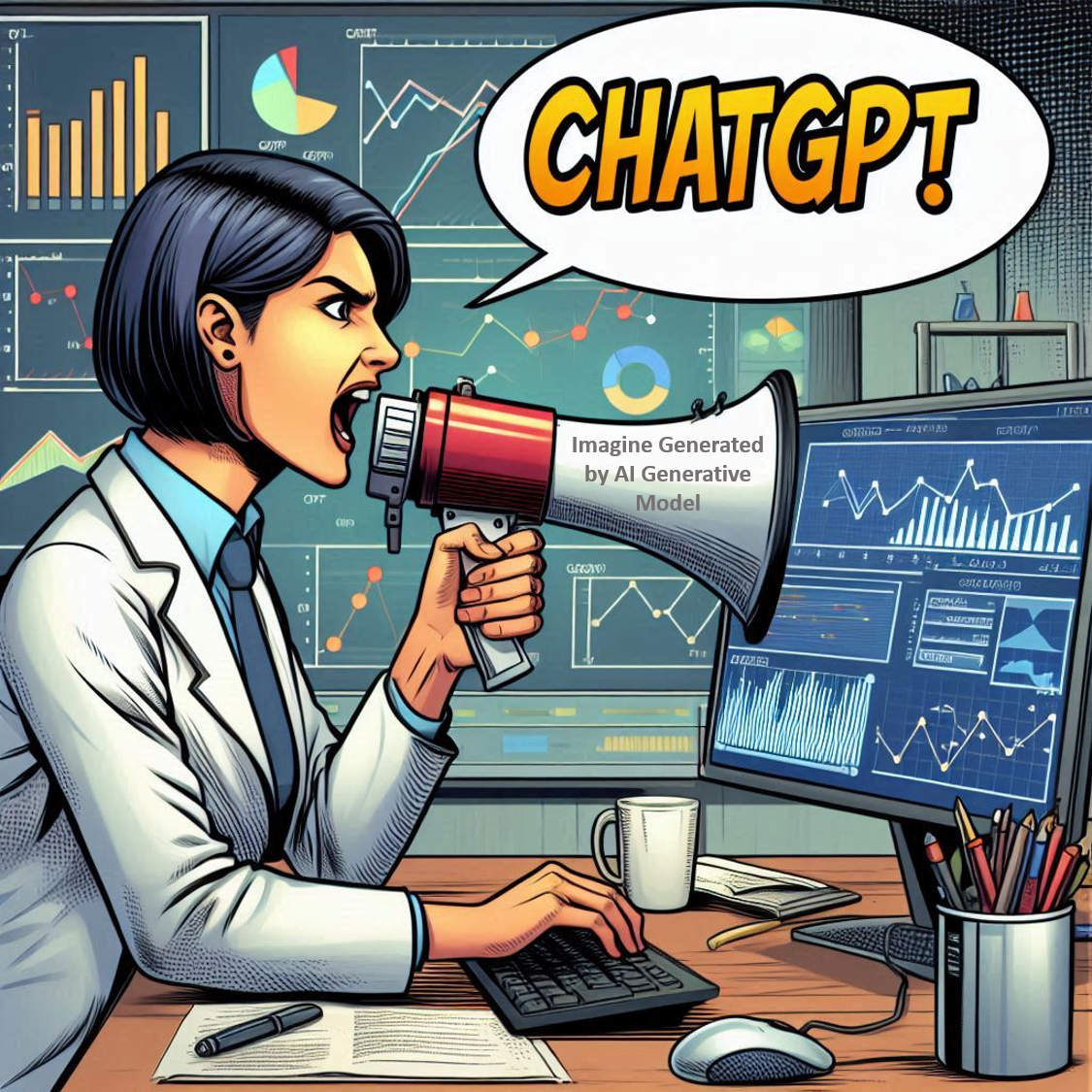

Supplement: Supplementary file 1 — Supplementary File contains a zip file. [file apb-15-467-s001.zip › Generative_AI_PKpop_Model-main/GraphicalAbstract.png]
